# Tumor-directed gene therapy in mice using a composite nonviral gene delivery system consisting of the *piggyBac *transposon and polyethylenimine

**DOI:** 10.1186/1471-2407-9-126

**Published:** 2009-04-27

**Authors:** Yu Kang, Xiaoyan Zhang, Wei Jiang, Chaoqun Wu, Chunmei Chen, Yufang Zheng, Jianren Gu, Congjian Xu

**Affiliations:** 1 Obstetrics and Gynecology Hospital, Fudan University, Shanghai 200011, PR China; 2 Department of Obstetrics and Gynecology of Shanghai Medical School, Fudan University, Shanghai 200011, PR China; 3 The State Key Laboratory of Genetic Engineering, School of Life Sciences, Fudan University, Shanghai 200433, PR China; 4 Department of Biophysics, School of Life Sciences, Fudan University, Shanghai 200433, PR China; 5 National Laboratory of Oncogene and Related Genes, Shanghai Cancer Institute, Shanghai 200032, PR China; 6 Institutes of Biomedical Sciences, Fudan University, Shanghai 200032, PR China

## Abstract

**Background:**

Compared with viral vectors, nonviral vectors are less immunogenic, more stable, safer and easier to replication for application in cancer gene therapy. However, nonviral gene delivery system has not been extensively used because of the low transfection efficiency and the short transgene expression, especially *in vivo*. It is desirable to develop a nonviral gene delivery system that can support stable genomic integration and persistent gene expression *in vivo*. Here, we used a composite nonviral gene delivery system consisting of the *piggyBac *(PB) transposon and polyethylenimine (PEI) for long-term transgene expression in mouse ovarian tumors.

**Methods:**

A recombinant plasmid PB [Act-RFP, HSV-tk] encoding both the herpes simplex thymidine kinase (HSV-tk) and the monomeric red fluorescent protein (mRFP1) under PB transposon elements was constructed. This plasmid and the PBase plasmid were injected into ovarian cancer tumor xenografts in mice by *in vivo *PEI system. The antitumor effects of HSV-tk/ganciclovir (GCV) system were observed after intraperitoneal injection of GCV. Histological analysis and TUNEL assay were performed on the cryostat sections of the tumor tissue.

**Results:**

Plasmid construction was confirmed by PCR analysis combined with restrictive enzyme digestion. mRFP1 expression could be visualized three weeks after the last transfection of pPB/TK under fluorescence microscopy. After GCV admission, the tumor volume of PB/TK group was significantly reduced and the tumor inhibitory rate was 81.96% contrasted against the 43.07% in the TK group. Histological analysis showed that there were extensive necrosis and lymphocytes infiltration in the tumor tissue of the PB/TK group but limited in the tissue of control group. TUNEL assays suggested that the transfected cells were undergoing apoptosis after GCV admission *in vivo*.

**Conclusion:**

Our results show that the nonviral gene delivery system coupling PB transposon with PEI can be used as an efficient tool for gene therapy in ovarian cancer.

## Background

Gene therapy is a promising strategy for the treatment of unresectable cancer. Non-viral gene transfer systems used in cancer gene therapy are attractive because they are relatively stable, safer, and easier to produce than viral vectors [1]. Conventional nonviral gene transfer systems include the direct tissue injection of DNA or transfection across the cell membrane using liposomes, peptide delivery systems, or polymer vectors[2]. Among them, the polycation polyethylenimine (PEI) have been extensively used for *in vivo *gene delivery [3]. PEI combines strong DNA compaction capacity with an intrinsic endosomolytic activity[4]. Recently studies showed that PEI derivatives can mediate efficient gene delivery in several tumors, such as lymphoma [5], hepatocellular carcinoma [6], pancreatic tumors [7], and ovarian carcinoma [8]. However, a significant drawback to PEI-mediated gene transfer delivery is that there still is no persistent transgene expression as there is no stable chromosomal integration [9]. The development of nonviral gene-transfer technologies that can support stable chromosomal integration and persistent gene expression *in vivo *is desirable.

Transposon system is a major step in overcoming that barrier as it is a technology that combines the advantages of nonviral delivery with genomic integration and persistent transgene expression. Transposon-derived sequences account for more than 40% of the genome in humans and mice [10,11], indicating the importance of transposition in evolution. Their ability to deliver and integrate specific genetic cargo into a host's chromosome has made transposons so useful as a molecular genetic tool in invertebrate species, and now they has progressively become more important as delivery vectors of therapeutic expression cassettes to treat human disease[12,13]. The main advantages of transposon-mediated genetic cargo delivery are: 1) Transgene in a chromosomally integrated transposon can be persistent expressed; 2) Transposon lacks viral sequences that could elicit unwanted immune or inflammatory responses [14]; 3) Some transposons have the capacity to accommodate large size foreign genes[15,16].

One of the well characterized DNA transposon vector used in mammals is the synthetic Sleeping Beauty (SB) transposon system. So far, SB transposon has been used successfully to mediate stable gene transfer and expression in several mouse and human cells [17-19]. However, as a transgene tool for therapeutic gene delivery in mammals, SB transposons was limited due to the facts that exhibits overproduction inhibition which limits in vivo activity [20] and that had a limited capacity to carry DNA fragments[21]. Recently, several other transposon vectors have been tested for their potential to deliver therapeutic genes, including Tol2[22], Frog Prince[23], and *piggyBac*(PB)[15,24-27]. These vectors differ in their efficiency of gene insertion, genetic cargo capacity, integration site preferences, and effects on chromosomal stability. Among them, PB, a functional DNA transposon from cabbage looper moth *Trichoplusia ni*, now was the widely used one in human gene therapy and mammalian mutagenesis. Wu et al. found that PB is the most active transposons compared with SB, Tol2 and Mos1. They also showed that PB lacks of overproduction inhibition, which is advantageous in preclinical development of transposon-based gene therapy[26].

Moreover, Ding and coworkers designed a binary cotransfection assay system consisting of both a donor plasmid containing the transposon and a helper plasmid containing the transposase. The donor plasmid, named PB [Act-RFP] DS, can carry multiple genes, which allows one to perform complex transgenic experiments such as identifying positive transgenic animals with the help of a visible marker. Their studies showed that cotransfection of donor plasmid and helper plasmid produced drug-resistant clones on average 50-fold higher than donor plasmid transfection alone, which confirmed that the enhanced clone production was due to trans-position. They suggested that the PB transposon can accommodate large foreign genes and readily mediate the introduction of foreign genes up to 14 kb in length. Therefore it can be an efficient transgenesis and insertional mutagenesis tool in mice and other vertebrate organisms[15].

In the present work, we adopted this PB transposon system carrying the herpes simplex thymidine kinase gene (HSV-tk gene) and delivered it to ovarian tumor grafted mice using PEI as the transfection reagent. The therapeutic potential of this nonviral gene delivery system was evaluated in an established mouse model of ovarian cancer. This is the first demonstration that PB combined with PEI as nonviral gene transfer system can be used as an efficient transgene tool for gene therapy in ovarian cancer.

## Methods

### Tumor cell lines

The cell line SKOV3 established from a human ovarian adenocarcinoma was obtained from the Shanghai Institute of Cell Biology of Chinese Academy of Sciences (Shanghai, China). Cells were grown in McCoy's 5A medium supplemented with 10% (V/V) fetal bovine sera, 100 U/mL of penicillin, and 100 mg/mL streptomycin (all purchased from GIBCO BRL, Grand Island, New York, USA). Cells were maintained at 37°C in a humidified incubator containing 5% CO_2 _and cultures were split twice per week.

### Plasmid construction

The plasmid PB [Act-RFP]DS (pPB), containing the mRFP1 expression cassette, and plasmid Act-PBase(pPBase), containing PB transposase were kindly provided by Xiaohui Wu (Institute of Developmental Biology and Molecular Medicine, School of Life Sciences, Fudan University, Shanghai, China). The plasmid pORF-HSVtk encoding the HSV-tk gene was obtained from Invivogen (San Diego, CA, USA). To generate the recombinant plasmid PB [Act-RFP, HSV-TK], named as pPB/TK, we first amplified HSV-tk cDNA using the forward primer 5'-ATTGGATCCGCTCCGGTGCCCGTCAGTGG-3' and the reverse primer 5'-CTAACTAGTTCAGTTAGCCTCCCCCATCTCC-3'. The PCR fragment was TA cloned into pMD18-T Simple vector (Takara, Dalian, China) and subcloned into *Bam*HI-*Spe*I sites of PB [Act-RFP] DS. Plasmid construction was identified by PCR analysis combined with restriction enzyme digestion. The primer sequences for HSV-tk were: forward primer, 5'-CCTGTGGTGCCTCCTGAACT-3'; reverse primer, 5'-GTTGCTATGGCCGCGAGAAC-3'. The predicted product size is 421 bp. For mRFP1, the forward primer was 5'-GGACGGCGAGTTCATCTACA-3', and the reverse primer was 5'-TTGACCTCGGCGTCGTAGTG-3', and the predicted product size is 191 bp.

### Tumor model

Animal study in this research has been performed with the approval of *Animal Ethics Committee of Obstetrics and Gynecology Hospital, Fudan University*. Female nude mice BALB/c at 6 weeks old were purchased from the Animal Center of the Chinese Academy of Science, Shanghai, China, and maintained in specific pathogen-free facility. All procedures were performed in accordance with *The Guide for the Ethical Treatment of Laboratory Animals *from the Ministry of Science and Technology of People's Republic of China (Publication No. 2006-398). To generate ovarian cancer tumor xenografts, SKOV3 cells (1 × 10^7 ^cells/0.1 ml of medium/mouse) were inoculated subcutaneously in the right flank of mice. Animals were inspected and weighed every three days.

### Complex formation and administration

*In vivo*-jetPEI was obtained from Polyplus-transfection (Illkirch, France). PEI/DNA complexes were prepared as follows: 3.2 μl of 150 mM PEI derivative in 5% glucose solution were vortex mixed with 10 μg of pPB/TK DNA and 10 μg of plasmid Act-PBase DNA in a final volume of 100 μl of 5% glucose solution. The glucosylated PEI/DNA mixture (N/P ratio = 8) was left for 15 minutes at room temperature, and then injected into xenografted mice intratumorally.

### Gene delivery to tumor in vivo

Transfection experiments were performed two weeks after tumor inoculation. Twenty mice were assigned to four groups: PB/TK treatment group (transfected with pPB/TK and pPBase), PB group (transfected with pPB and pPBase), TK group (transfected with pORF-HSVtk) and control group (5% (vol/vol) dextrose). Each group contained an equal number of large and intermediate-sized tumors so that the mean tumor volume in each group was comparable before intratumoral injection. Every mouse was intratumorally injected with 100 μl PEI/DNA complexes once a day on days 1, 4 and 7. Twenty-four hours after the first transfection, 25 mg/kg ganciclovir (GCV) was injected intraperitoneally twice daily for 7 days. Tumor size was measured with calipers two times per week, and tumor volume was estimated from two-dimensional tumor measurements using the formula: V = length (mm) × width^2 ^(mm^2^)/^2^. Three weeks after the last transfection *in vivo*, mice were euthanized and necropsies were performed. The weights of tumors were measured to determine the tumor weight (g) and inhibitory rate (%). The tumor inhibitory rate was calculated using the following formula: Inhibitory rate = [(tumor weight of control group-tumor weight of experiment group)/tumor weight of control group] × 100%. After measuring final tumor weight, all tumors and major organs, such as heart, lung, liver, spleen, kidney, were fixed in formalin and embedded in paraffin for further sections. Histopathological analysis was performed following routine hematoxylin and eosin (H&E) staining on sections.

### Assaying transgene expression

To monitor transgene expression, the mRFP1 reporter gene was assessed in frozen tumor sections. Three weeks after the last transfection of pPB/TK and intraperitoneal injection of GCV, mice were scarified and formaldehyde-fixed 6-μm frozen sections were rinsed in PBS and mounted on glass slides. mRFP1 expression was visualized using fluorescence microscopy (Nikon Eclipse E600, Tokyo, Japan). Empty vector pPB combined with PEI was used as control.

### Immunofluorescence costaining

To examine the apoptosis of transfected cells, which was induced by GCV *in vivo*, cryostat sections of tumors were fixed with acetone and analyzed for TUNEL-positive cells using a fluorescence *in situ *cell death detection kit (Roche Molecular Biochemicals, Indianapolis, IN) according to the manufacturer's instructions. In brief, sections were treated with the TUNEL reaction mixture (including FITC-conjugated dUTP) and incubated in the dark for 1.5 h at 37°C, followed by PBS washes. Sections were examined using a fluorescence microscope.

### Statistical analysis

Results are expressed as means ± SD. One-way ANOVA was used to evaluate differences between groups, and a *p *value less than 0.05 was considered significant.

## Results

### Recombinant plasmid construction

**Figure 1:**
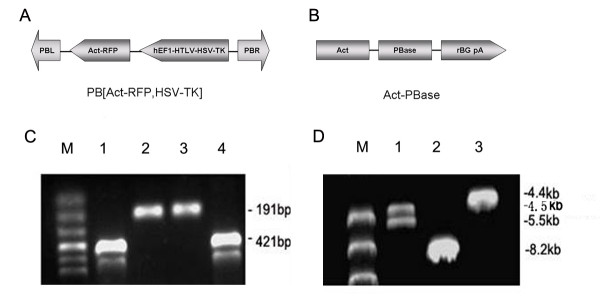
**Construction of recombinant Plasmid PB [Act-RFP, HSV-tk]**. (A) PB [Act-RFP, HSV-tk] constructs. The HSV-tk gene was cloned in the *BamHI-SpeI *sites of the PB [Act-RFP] DS (pPB), generating PB [Act-RFP, HSV-tk] (pPB/TK), which consists of both HSV-tk and mRFP1 expression cassettes. PBL and PBR was a pair of PB repeat termini; (B) PB transposase helper constructs. The *piggyBac *transposase gene (PBase) driven by β-actin (Act) promoters was followed by rabbit β-globin polyA (rBG pA) (adapted from Ref [15]); (C) PCR analysis of pPB/TK: Lane 1: Marker; Lane 2: pPB/TK: PCR product of tk fragment; Lane 3: pPB/TK: PCR product of mRFP1 fragment; Lane 4: pPB: PCR product of mRFP1 fragment; Lane 5: pORF-HSVtk: PCR product of tk fragment; (D) Restriction analysis of pPB/TK: Lane 1: Marker; Lane 2: pPB/TK; Lane 3: pPB; Lane 4: pORF-HSVtk.

The HSV-tk gene was cloned within the *Bam*HI-*Spe*I sites of the PB transposon pPB, generating recombinant plasmid pPB/TK (10 kb) containing both mRFP1 and the HSV-tk expression cassettes in Figure 1A. Figure 1B illustrates the helper plasmid which contains a PB transposase gene (PBase) driven by β-actin (Act) promoters and followed by rabbit β-globin polyA (rBG pA). Figure 1C shows positive pPB/TK clones confirmed by PCR amplification of the HSV-tk and mRFP1 genes fragments at 421 bp and 191 bp, respectively. We also confirmed the positive clone by restriction digest with *Hin*dIII. The correct clones of pPB/TK generated two fragments at 5.5 kb and 4.5 kb, while digestion of pPB or pORF-HSVtk yielded a single band at 8.2 kb and 4.4 kb, respectively (Figure 1D).

### Transposition mediates transgene expression in vivo

**Figure 2:**
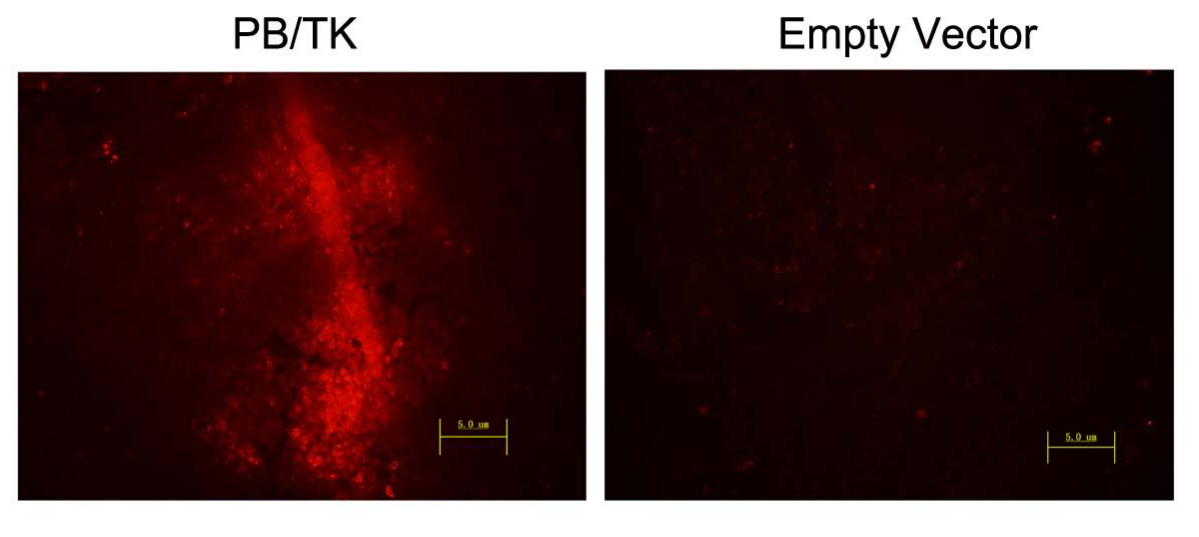
**Transgene expression in vivo**. Fluorescence micrographs of pPB/TK-transfected tumor cryosections show that mRFP1 expression was observed three weeks after transfection. Empty vector was used as control. (Magnification, ×200).

As mRFP1 is a marker for transgene expression, so mRFP1 expression in tumors was examined to ascertain whether PB transposon-mediated gene transfer supports stable gene expression in mice. The results indicated that the mRFP1 expression could be observed even three weeks after transfection on pPB/TK-transfected tumor cryosections, but not in empty vector controls (Figure 2).

### Tumor growth inhibition after HSV-TK gene transfer and GCV administration

**Figure 3:**
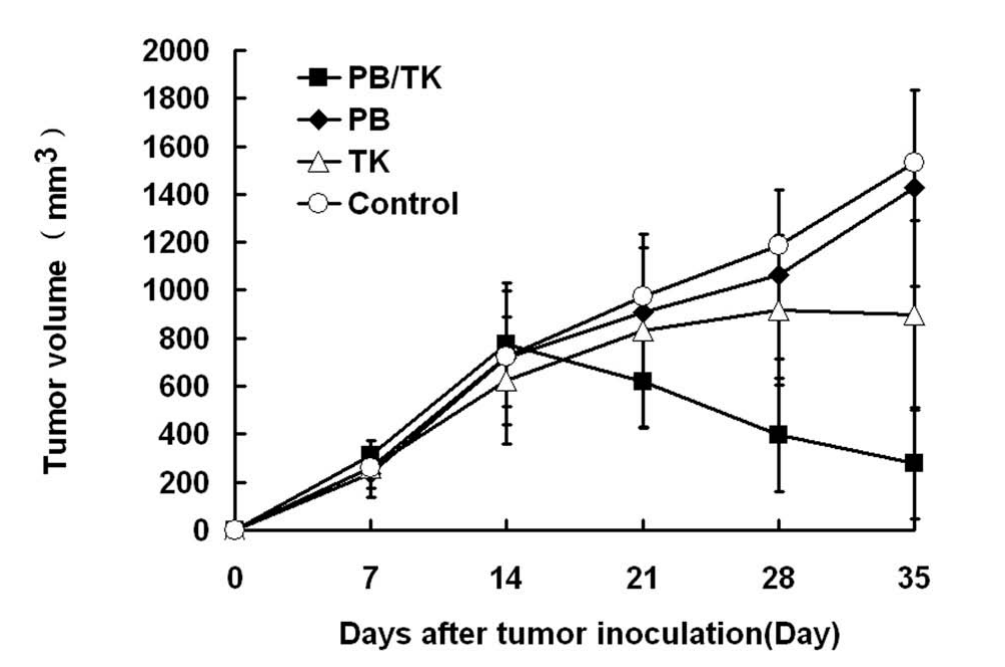
**Tumor growth inhibition assay**. Two weeks after subcutaneous inoculation of SKOV3 cells in BALB/c nude mice, complexes of pPB/TK, pPB, pORF-HSVtk with PEI were injected directly into the tumor three times (days 1, 4 and 7). GCV solution was administered by daily intraperitoneal injection beginning on the day after the first complex injections. Tumor volumes were monitored over time. Mice were sacrificed 3 weeks after transfection in vivo. Errors bar represent the standard error of the mean.* p < 0.01, comparing the PB/TK group with the control group at day 21.

To test the *in vivo *sensitivity of the pPB/TK transfected cells to GCV, the mice were cotransfected with pPB/TK and pPBase using PEI, and subsequently treated with GCV. Figure 3 shows that the tumor volume of PB/TK group was significantly reduced, while much less tumor volume was observed in the TK group. At day 21, the tumor inhibitory rate in PB/TK group was 81.96% contrast to 43.07% in the TK group (*p *< 0.01, Table 1). No difference in the rate was observed between the PB group and control group. In all animals, no noticeable toxicity was observed as no obvious damage on major organs was discovered by histopathological analysis (data not shown). The results demonstrate that nonviral gene transfer by PB transposon combined with PEI might result in potent tumor growth inhibition.

**Table 1:** Tumor weight on the 21st day after transfection in vivo

Group	Weight of tumor (X ± SD, g)	Tumor inhibitory rate (w/w,%)
**PB/TK**	0.31 ± 0.23	81.96 *
**PB**	1.61 ± 0.41	6.40
**TK**	0.98 ± 0.36	43.07
**Control**	1.72 ± 0.54	

Growth inhibition was observed in the PB/TK group compared with the other groups. *p < 0.01, comparing the PB/TK group with the control group.

### Histological Analysis of different subgroups

**Figure 4:**
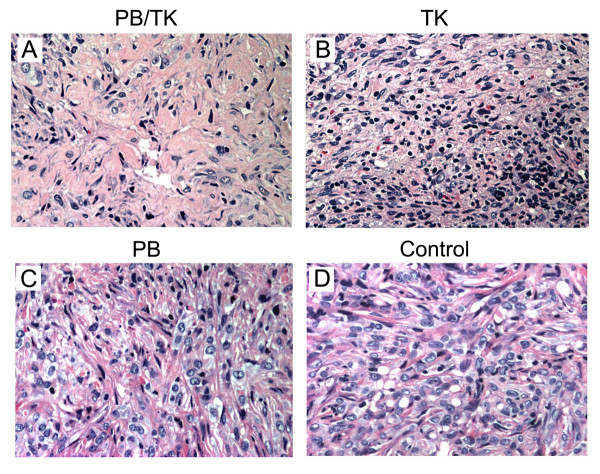
**H&E staining of tumor tissue samples removed from different subgroups**. PB/TK group: Extensive necrosis; TK group: Minimal necrosis but moderate lymphocyte aggregation around tumor cells; PB group & Control group: Enlarged hyperchromatic and pleomorphic nuclei with irregular nuclear membrane and nucleoli. (Magnification, ×200).

To determine the antitumor efficacy of the HSV-tk/GCV system *in vivo*, paraffin-embedded tissues were sectioned at 4 μm and stained with H&E. The sections from the tumor tissue of the PB/TK group showed extensive necrosis and lymphocyte infiltration, as shown in figure 4A. Meanwhile, minimal necrosis and moderate lymphocyte infiltration were identified in the TK group sections (Figure 4B). Necrosis was absent in tumor samples from both the PB and control groups (Figure 4C, D).

### In vivo killing of transfected cells by GCV

**Figure 5:**
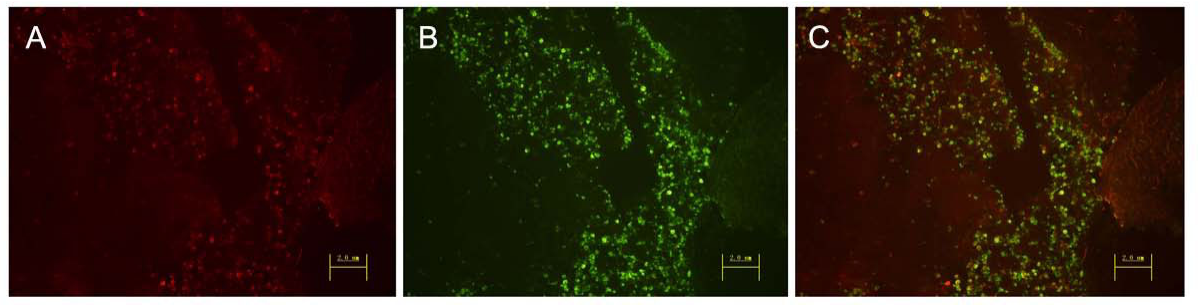
**In vivo sensitivity of the HSV-tk transfected cells to GCV (Magnification, ×200)**. (A) The PB/TK transfected cells are shown as bright red. (B) Apoptotic cells are bright green after staining with TUNEL assay kit. (C) The merged cells (yellow) suggest the transfected cells were undergoing apoptosis after GCV treatment *in vivo*.

To evaluate the killing mechanism of transfected cells treated with GCV, TUNEL assays were performed on mouse tumor sections. Transfected cells were shown as bright red with mRFP1 expression (Figure 5A), and apoptotic cells were labeled with bright green (Figure 5B). The merged image (yellow) suggested that almost all transfected cells were undergoing apoptosis after GCV treatment *in vivo *(Figure 5C).

## Discussion

Ovarian carcinoma is a common cancer worldwide, and there is still no effective therapy for such unresectable disease. Gene therapy may represent an attractive alternative to the classical treatments, and numerous experimental approaches have shown potential promise in treatment of ovarian carcinoma [28-30]. Among them, several protocols are based on the use of suicide genes such as the HSV-tk gene, which can induce significant reduction in tumor burden and can prolong survival after the prodrug GCV treatment. However, the clinical application of this approach may be hindered by transgene vectors. Current viral vectors carry a risk for generating active viral particles through recombination, and triggering unwanted host inflammatory and immune responses [31,32], while nonviral vectors lack the ability to induce stable chromosomal integration and persistent gene expression *in vivo *[33].

In recent years there has been an increased understanding of the mechanisms behind transgene delivery. Successful delivery of foreign genes to a mammalian cell involves several discrete steps: delivery of genes to target cells, cellular entry, endosome escape, and integration into the nucleus [34,35]. To date, no single gene transfer system has been developed that can effectively fulfill the requirements for all of the features described above. A combination of complementary, multi-functional nonviral gene transfer systems may satisfy the major requirements for efficient gene delivery.

Cationic gene carriers have a net positive charge and can facilitate the cellular entry of DNA by interacting with the negatively charged cell surface [35,36]. Endosomal escape is achieved by including components that induce membrane fusion at low pH or block pH lowering inside the endosome in the DNA-carrier complex. PEI is a cationic polymer that serves as an effective vehicle for *in vivo *gene delivery in many tissues. It facilitates effective DNA binding and protection, combined with the capability to escape the endosome due to its proton buffering ability with resultant osmotic swelling and endosomal disruption [36]. PEI has been shown to be an efficient transfection agent for ovarian carcinoma cells by Intraperitoneal injection [8]. However, several studies indicated that PEI-mediated transgene expression was dose-dependent and transient, with maximal transgene expression observed during the 48-hour period immediately following transfection [37]. Very low amounts of DNA remained detectable after 72 h, indicating probable degradation and clearance [8]. Thus, the development of nonviral gene-transfer technologies that can support stable chromosomal integration and persistent gene expression *in vivo *is desirable.

Transposon-mediated DNA delivery has opened the door to the development of a new generation of vectors for human gene therapy and mammalian [12]. Transposon-based systems have been proven to be effective integrating nonviral vectors that can mediate long-term *in vivo *transgene expression [17]. Ohlfest and coworkers used SB transposons as an intratumoral gene transfer vector for glioblastoma multiforme, and delivered using PEI as gene transfer reagent. They observed marked anti-tumor activity as demonstrated by reduced tumor vessel density, inhibition of tumor growth, and tumor elimination in up to 50% of nude mice[38,39]. In the present study, we used PB, a more active transposon[15,24-27], combined with PEI to deliver HSV-TK gene into ovarian cancer xenografts. Our results demonstrate that the expression of foreign DNA mediated by PB transposon and PEI is much more efficient than PEI alone. After HSV-TK gene transfer and GCV administration, significant tumor growth inhibition in vivo was observed up to 82%. The tumor inhibition rate is much higher than previous study by SB mediated gene therapy, probably due to PB transposon is more efficient than SB transposon. Long-term expression and bystander effect of HSV-TK gene may contribute to the result. However, we still do not know the exact mechanism how PB transposon could cause more tumor inhibition effect *in vivo*. Further studies should be considered to determine how PB transposon performs *in vivo *in long term studies. Furthermore, for PB transposon therapy to transition into clinical consideration, the potential safety issues from insertional disruption or deregulation in genome will also need to be addressed.

## Conclusion

In summary, our results demonstrate the feasibility and effectiveness of a nonviral gene transfer system combinding the PB transposon with PEI. This system has shown great efficiency on treating ovarian caner in our model, however, the therapeutic effect of our method against other carcinoma needs further investigation.

## Abbreviations

PB transposon: *piggyBac *transposon; PEI: polyethylenimine; HSV-tk: herpes simplex thymidine kinase; mRFP1: monomeric red fluorescent protein; GCV: ganciclovir; TUNEL: terminal deoxynucleotidyl transferase dUTP nick end labeling; SB transposon: Sleeping Beauty transposon; PCR: polymerase chain reaction.

## Competing interests

The authors declare that they have no competing interests.

## Authors' contributions

YK helped design some of the experiments, performed the *in vitro *and *in vivo *studies and drafted the manuscript; XYZ and WJ carried out the animal studies; CQW and CMC provided technical assistance throughout the study and analyzed the data; JRG and YFZ helped design the experiment and participated in completing manuscript. CJX conceived the study, and participated in its design and coordination. All authors read and approved the final manuscript.

## Pre-publication history

The pre-publication history for this paper can be accessed here:

http://www.biomedcentral.com/1471-2407/9/126/prepub
